# Conformational pathway provides unique sensitivity to a synaptic mGluR

**DOI:** 10.1038/s41467-019-13407-8

**Published:** 2019-12-05

**Authors:** Chris H. Habrian, Joshua Levitz, Vojtech Vyklicky, Zhu Fu, Adam Hoagland, Isabelle McCort-Tranchepain, Francine Acher, Ehud Y. Isacoff

**Affiliations:** 1 Biophysics Graduate Group, University of California, Berkeley, CA 94720 USA; 2 Department of Molecular and Cell Biology, University of California, Berkeley, CA 94720 USA; 3 Department of Biochemistry, Weill Cornell Medical College, New York, NY 10024 USA; 4 Paris Descartes University, 75006 Paris, France; 5 Helen Wills Neuroscience Institute, University of California, Berkeley, CA 94720 USA; 6 Molecular Biology & Integrated Bioimaging Division, Lawrence Berkeley National Laboratory, Berkeley, CA 94720 USA

**Keywords:** Biophysics, Neuroscience

## Abstract

Metabotropic glutamate receptors (mGluRs) are dimeric G-protein–coupled receptors that operate at synapses. Macroscopic and single molecule FRET to monitor structural rearrangements in the ligand binding domain (LBD) of the mGluR7/7 homodimer revealed it to have an apparent affinity ~4000-fold lower than other mGluRs and a maximal activation of only ~10%, seemingly too low for activation at synapses. However, mGluR7 heterodimerizes, and we find it to associate with mGluR2 in the hippocampus. Strikingly, the mGluR2/7 heterodimer has high affinity and efficacy. mGluR2/7 shows cooperativity in which an unliganded subunit greatly enhances activation by agonist bound to its heteromeric partner, and a unique conformational pathway to activation, in which mGluR2/7 partially activates in the Apo state, even when its LBDs are held open by antagonist. High sensitivity and an unusually broad dynamic range should enable mGluR2/7 to respond to both glutamate transients from nearby release and spillover from distant synapses.

## Abstract (web summary)

Metabotropic glutamate receptors (mGluRs) are dimeric G-protein–coupled receptors that operate at neural synapses. Here authors use FRET assays in live cells to monitor mGluR2/7’s activation and reveal how heterodimerization can alter the glutamate response of an mGluR.

## Introduction

G-protein–coupled receptors (GPCRs), the largest class of membrane signaling proteins, respond to a wide array of extracellular stimuli to initiate intracellular signaling via G proteins and arrestins^1^. Recent studies have provided snapshots of GPCR structures in distinct conformations^2–5^ and revealed that they are extremely dynamic^6–13^. The conformational dynamics appear to be central to ligand recognition, activation and signaling by GCPRs^9,14,15^.

Membrane receptors have evolved to respond to precise spatio-temporal concentration profiles of extracellular ligands. In the nervous system, neurotransmitter receptors encounter a wide range of neurotransmitter concentrations and spatio-temporal profiles. Key factors contributing to these dynamics are the small extracellular volume of the synaptic cleft, pumps and/or enzymes that remove neurotransmitter and diffusion. Additionally, neurotransmitter receptors can be localized within the synapse both pre- and postsynaptically as well as extrasynaptically where they can encounter different sources of neurotransmitter released either locally, which briefly reaches low millimolar levels within a synaptic cleft, and spillover from nearby synapses, which reaches lower concentrations^16^. Thus, receptor activation by transmitter needs to be tuned to suit its localization and sensitivity to transmitter. GPCRs have evolved to respond to diverse signal types at various cellular locations and produce a large variety of responses. Structural and functional diversity in receptor families can provide unique responses in ligand activation and localization, as can specialized post-translational modification or interaction with regulatory proteins^3,17,18^.

Most synaptic receptors have apparent affinities (EC50s) in the low micromolar range, or lower^19–21^. One intriguing outlier is metabotropic glutamate receptor 7 (mGluR7). This receptor, one of an 8-member family of class C GPCRs^22^, has been reported, based on effector activation, to have an apparent affinity that is much lower than that of the other mGluRs^23^. These properties have led to the idea that mGluR7 may only function at locations very near the site of glutamate release^24^, and best at synapses with high repetitive activity and enclosed extracellular volumes^25^. Even under these conditions, it is not clear how effectively mGluR7 is activated.

mGluRs are verified targets of numerous neurological disorders and are considered to contain great potential as therapeutic targets^26–28^, unfortunately many years of work have resulted in no mGluR targeting drugs approved for medical use at the present time. Recent work has, for the first time, revealed structures of the active and inactive conformations allowing for more detailed understanding and interpretation of the molecular mechanism of mGluR activation^29^. mGluRs are strict dimers^30,31^ and dimerization is required for efficient G protein activation^32^. Heterodimerization can occur between group I members mGluR1 and 5 and within and between members of group II (mGluR2 and 3) and group III (mGluR4, 6, 7, and 8)^30,31^. Functional analysis of one such potential combination, mGluR2/mGluR3 (mGluR2/3), showed that heterodimerization can impart unique functional properties resulting in distinct basal activity^31^. We wondered if heterodimerization of mGluR7 would increase its activation by glutamate. While, overlapping expression suggests that mGluR7 may be co-expressed with other mGluRs and therefore might heterodimerize in the brain, only one example of heterodimer formation has been demonstrated to date in neurons, that of mGluR2/4^33,34^. But the mGluR2/4 heterodimer resembles its parents functionally, not surprisingly since they themselves have similar activation in response to glutamate^34^.

To analyze the activation of the mGluR7/7 homodimer, we fluorescently labeled with donor and acceptor Foster resonance energy transfer (FRET) dyes a protein tag fused to the mGluR7 N-terminal, immediately before the ligand binding domain (LBD). We measured FRET changes between the LBDs of the dimer, which result from the conformational rearrangements of activation, in which the receptor dimer transitions from a state with both LBDs empty and open and the receptor at rest (Roo) to a state in which both LBDs are occupied by agonist and closed and the receptor is activated (Acc)^11,13,30,31,35^. Measurements in live HEK 293T cells and at the single-molecule level on immune-purified dimers^13,31^ confirm very low affinity^23^ and efficacy^36^. We confirm and extend these findings measuring an apparent affinity of 38 mM and a max efficacy of 10%, values too low, it would appear, to support activation by the concentrations estimated for synaptic glutamate^37,38^. However, we find that mGluR7 associates with mGluR2 in hippocampus and that the mGluR2/7 heterodimer has even higher affinity and efficacy than mGluR2/2, a wider dynamic range, showing that heterodimerization can alter the glutamate response of an mGluR. The boosted properties are associated with a special heteromeric cooperativity that boosts the affinity and efficacy of each subunit in the dimer, and which primes the receptor for activation even in absence of agonist through a unique tendency of the LBDs to enter the rotated activated conformation even when they are in the open, Apo state. Combined with unusually fast kinetics, these properties appear to make mGluR2/7 uniquely suited to activate in response to synaptic glutamate.

## Results

### mGluR7 activates with very low affinity and efficacy

**Fig. 1:**
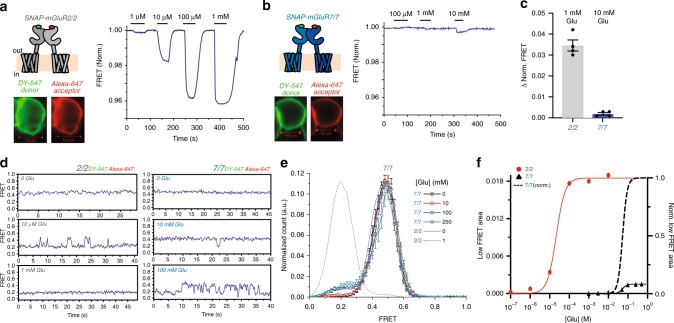
Glutamate activates mGluR7/7 with low affinity and low efficacy. **a**, **b** Homodimers of SNAP-tagged mGluR2/2 (**a**) and mGluR7/7 (**b**). Left) Schematics (top) and images of expressing HEK293 cells labeled with a mixture of donor (green) and acceptor (red) dyes. Right) Live-cell FRET traces show responses to ascending concentrations of glutamate. Scale bars are 5 µm. **c** Mean + S.E.M. fractional change in FRET of SNAP-mGluR2/SNAP-mGluR2 (1 mM glutamate, *n* = 3) and SNAP-mGluR7/SNAP-mGluR7 (10 mM glutamate, *n* = 3, *p *<* 0.05). Individual data points (black dots) and s.e.m. (error bars). **d** Representative smFRET traces of SNAP-mGluR2/SNAP-mGluR2 (left) and SNAP-mGluR7/SNAP-mGluR7 (right) at different glutamate concentrations. **e** Histogram of smFRET distributions of SNAP-mGluR7/SNAP-mGluR7 in 0 (5 movies, 288 molecules), 10 mM (6 movies, 215 molecules), 100 mM (5 movies, 261 molecules) and 250 mM (5 movies, 253 molecules) glutamate concentrations (colored symbols and interpolation lines, error bars are s.e.m.) and of SNAP-mGluR2/2 in 0 (5 movies, 230 molecules) and 1 mM (4 movies, 197 molecules) glutamate (solid and broken gray lines, respectively). **f** Titration curve of low smFRET (activated state) peak in histograms of SNAP-mGluR2/SNAP-mGluR2 (red circle) and SNAP-mGluR7/SNAP-mGluR7 (black triangle; normalized as black dashed line). Donor (BG-DY-547) and acceptor (BG-Alexa 647) dyes imaged at 10 Hz for ensemble FRET in cells (**a**–**c**) and 10 Hz for smFRET on the isolated protein (**d**–**f**).

To understand the unusually low apparent glutamate affinity of mGluR7/7 observed earlier^23^, we compared the ligand-induced conformational rearrangement of mGluR7/7 with that of mGluR2/2. As previously described^13,30^, N-terminally SNAP-tagged mGluR2 expressed in HEK293T cells and labeled with Alexa-647 (acceptor) and DY-547 (donor) fluorophores (Förster radius 52 Å) showed a robust glutamate-induced FRET decrease, which increased with glutamate concentration between 1 µM and 1 mM (Fig. 1a), with an EC50 of 18.7 + 0.6 µM (Supplementary Fig. 1a). A similarly N-terminally SNAP-tagged mGluR7, expressed at comparable levels, as assessed from SNAP dye staining, showed no detectable FRET change at up to 1 mM glutamate, and a barely measurable FRET decrease at 10 mM glutamate (Fig. 1b), which was >30-fold smaller than that observed at 1 mM glutamate in mGluR2 (Fig. 1c). To test whether interaction with a signaling complex affected activation, Gβγ-activated GIRK1 inward rectifier potassium channel was co-expressed with the receptor. We observed no change in the glutamate-induced FRET response of either SNAP-mGluR2 (Supplementary Fig. 1a) or SNAP-mGluR7 in the presence of GIRK channels (Supplementary Fig. 1b). Nor did a switch to another donor/acceptor dye pair, which gave robust glutamate-induced FRET responses in SNAP-mGluR2 make for detectable responses in SNAP-mGluR7 (Supplementary Fig. 1c). In contrast to SNAP-mGluR7, a SNAP-tagged version of another group III mGluR, mGluR4, which has been shown to have low micromolar apparent glutamate affinity, showed glutamate-induced FRET decreases that were similar in amplitude and concentration dependence to those of mGluR2 (Supplementary Fig. 1d), and similar to those reported earlier for several of the other mGluRs^13,30^.

In HEK293T cells it was not possible to go to glutamate concentrations above 10 mM. We therefore turned to isolated receptors. Receptors expressed in HEK293T cells were labeled with a mixture of SNAP-reactive donor and acceptor fluorophores, detergent solubilized, immune-purified and tethered by biotinylated secondary antibodies at low density to coverslips that were passivated with a lawn of polyethylene glycol^13^. We used single-molecule FRET (smFRET) in order to measure the absolute FRET levels and dynamic changes associated with ligand-induced conformational changes in the LBDs. We imaged the donor and acceptor dyes using total internal reflection fluorescence (TIRF) microscopy^13,31^ (Supplementary Fig. 1e).

Our baseline for comparison was mGluR2. As previously observed^13^, in zero glutamate, the two unliganded LBDs of mGluR2 are both open and in the resting configuration (Roo) and the inter-subunit distance between donor and acceptor SNAP dyes is relatively small, giving a high FRET level of ~0.45, which can be seen in single-molecule trajectories from an individual dimer (Fig. 1d, left top) and in histograms that compile measurements from many dimers (Fig. 1e, solid gray line). At 1 mM glutamate, the LBDs of mGluR2 are maximally bound, closed and rotated into the activated conformation (Acc) to yield a low FRET level of ~0.2 (Fig. 1d, left bottom; Fig. 1e, dashed gray line). At an intermediate glutamate concentration of 10 µM, near the EC50 of the ensemble concentration-response FRET measured both in live HEK293T cells (Supplementary Fig. 1a) and on isolated protein in smFRET (Fig. 1f, red symbols), mGluR2 transitions between the high and low FRET states (Fig. 1d, left middle), yielding high conformational dynamics, as measured by donor-acceptor cross-correlation (Supplementary Fig. 1f, left).

Similar to mGluR2, mGluR7 in zero glutamate had a stable FRET level of ~0.45 (Fig. 1d, right top; Fig. 1e, black symbols). Remarkably, at 10 mM glutamate (10-fold higher than the saturating concentration tested in mGluR2), only rare transitions were seen in mGluR7 from the high FRET state to a low FRET state of ~0.2 (Fig. 1d, right middle). While the low FRET level was similar to that of mGluR2, in mGluR7 these events were so short-lived and infrequent that a low FRET peak was barely observable in the pooled histogram (Fig. 1e, red symbols). The low FRET peak increased with increasing concentration, reaching a maximum between 100 and 250 mM (Fig. 1d, right bottom; Fig. 1e, blue and cyan symbols; Fig. 1f; Supplementary Fig. 1g,h). The EC50 of the mGluR7 low FRET peak concentration-response was 38.3 mM, an apparent glutamate affinity that is ~4,000-fold lower than that of mGluR2 (Fig. 1f). Single-molecule trajectories showed that, although at saturating glutamate (≥100 mM) transitions to the low FRET state occurred more frequently than at 10 mM glutamate, the maximal occupancy of the glutamate activated state only reached ~10% (Fig. 1f). The low degree of maximal activation indicates that glutamate is a low efficacy partial agonist of mGluR7.

### Increasing mGluR7 efficacy

**Fig. 2:**
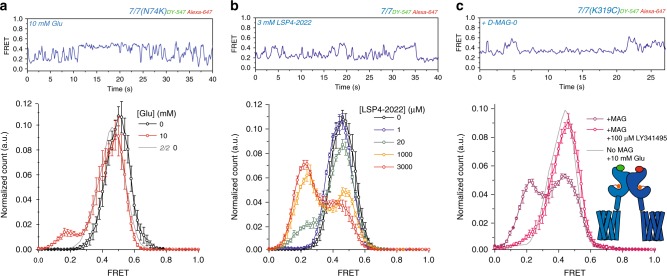
Increasing mGluR7 efficacy. **a**–**c** Representative smFRET traces (top) and smFRET histograms (bottom) of homodimers of SNAP-mGluR7(N74K) (0 Glu: 4 movies, 124 moleucles, 10 mM Glu: 4 movies, 140 molecules, s.e.m. error bars) compared to SNAP-mGluR2 in glutamate (**a**); SNAP-mGluR7 in LSP4-2022 (0: 4 movies, 145 molecules, 1 μM: 5 movies, 197 molecules, 20 μM: 5 movies, 128 molecules, 1 mM: 5 movies, 122 molecules, 3 mM LSP4: 6 movies, 206 molecules, s.e.m. error bars) (**b**); and SNAP-mGluR7(K319C) conjugated to D-MAG-0 in the *trans* configuration (8 movies, 230 molecules, s.e.m error bars), in the presence of 100 μM LY341495, and unconjugated SNAP-mGluR7(K319C) (6 movies, 256 molecules, s.e.m. error bars) (**c**, and cartoon insert). Donor (BG-DY-547) and acceptor (BG-Alexa 647) dyes imaged at 10 Hz.

To pursue the surprising observation that glutamate is such a low efficacy agonist of mGluR7, we asked whether a point mutation in the LBD agonist binding pocket, N74K, which has been shown to increase apparent affinity of mGluR6 and mGluR7^36^, would also affect efficacy. Measurements in smFRET showed that 10 mM glutamate induces more frequent activation transitions in mGluR7(N74K) (Fig. 2a, top) and greater occupancy of the activated low FRET (~0.2) state (Fig. 2a, bottom) than seen in wild-type mGluR7 at >10X higher glutamate (Fig. 1e). This reflects an increase in the stabilization of glutamate binding within the LBD.

We next asked if it was possible to increase agonist binding and occupancy at the binding site of wild-type mGluR7 using a synthetic agonist. We turned to a new synthetic group III selective agonist, LSP4-2022, which is highly selective for mGluR4, activating it efficiently at nanomolar concentration^39,40^. We found that LSP4-2022 is a potent activator of mGluR7 at higher concentrations. At 20 μM LSP4-2022, smFRET traces showed frequent transitions to the low FRET activated conformation, and occupancy of the low FRET conformation reached ~65% at 3 mM LSP4-2022 (Fig. 2b and Supplementary Fig. 2a), the highest concentration we could test, indicating an at least 6-fold greater efficacy than that of glutamate (compare Figs. 1e and 2b).

We next asked whether glutamate itself could be turned into a more potent agonist of mGluR7 if the glutamate were lodged stably into the LBD binding pocket. To achieve this, we used a photoswitchable tethered glutamate, maleimide-azobenzene-glutamate D-MAG-0 (Supplementary Fig. 2b), which attaches covalently to the LBD and docks its glutamate into the agonist binding pocket in mGluRs in one of the photo-isomeric configurations of azobenzene, achieving a high effective concentration^31,41,42^. When conjugated to an engineered cysteine on the lower lobe of the mGluR7 LBD (K319C), D-MAG-0 activated mGluR7 in the *trans* configuration of azobenzene (in the dark and under ~500 nm light), and deactivated in the *cis* configuration (~380 nm light), as measured by activation of the G protein activated inward rectifier potassium channel, GIRK1(F137S) (Supplementary Fig. 2c, d). The K319C mutation did not alter the apparent affinity of mGluR7 for glutamate (Supplementary Fig. 2e). Thus, D-MAG-0 is an agonist of mGluR7 in the *trans* configuration of azobenzene. This enabled us to perform FRET experiments to monitor the activation rearrangement of the LBD and photoswitch D-MAG-0. We used illumination at 532 nm to simultaneously excite the FRET donor and photo-isomerize D-MAG-0 into the agonistic *trans* state.

smFRET was performed on purified SNAP-mGluR7(K319C) homodimers that were labeled with donor and acceptor dyes on the SNAP and D-MAG-0 on K319C in the D-MAG-0 activated state. The smFRET trajectories showed frequent transitions into the low FRET activated state (Fig. 2c, top). Histograms that pooled the behavior of many dimers showed that the occupancy of the activated low FRET state was ~50% (Fig. 2c, bottom). Addition of the high affinity orthosteric antagonist LY341495 caused a nearly complete disappearance of the low FRET peak (Fig. 2c, bottom), consistent with displacement of the glutamate of D-MAG-0 from the orthosteric binding site. These observations show that the tethered glutamate of D-MAG-0 stabilizes the activated conformation of mGluR7 approximately 5-fold more effectively than does saturating free glutamate. This suggests that the low efficacy of glutamate in mGluR7 may result from a mismatch between the kinetics of glutamate binding and unbinding and the kinetics of LBD closure/activation rotation, which are overcome when *trans* D-MAG-0 jams its glutamate into the ligand binding pocket.

### mGluR7 heterodimerization with mGluR2

Our observations, so far, suggest that mGluR7 has an active state conformation that is similar to that of other mGluRs, that this conformation is only weakly stabilized by glutamate, and that pointing glutamate into the binding pocket on a stiff tether boosts efficacy, indicating that mGluR7 is capable of strong activation by glutamate. We wondered whether some modification of mGluR7 could change its properties so that it would be more strongly activated by glutamate.

It is known that mGluR7 can heterodimerize with other mGluRs^30^ and earlier work suggests that mGluR7 expression may overlap with that of other mGluRs in the mammalian brain^43^. We wondered, therefore, if mGluR7 heterodimerization occurs in neurons and, if so, whether a heterodimer containing mGluR7 would have stronger activation by physiological levels of glutamate. mGluR7 is expressed strongly in the hippocampus, olfactory bulb and tubercule, and cortex^44^. In the hippocampus, other mGluRs are also expressed, including group I members mGluR1 and 5 and group II members mGluR2 and 3, with the highest expression of mGluR5 and 2^45^. Earlier studies showed that mGluRs co-assemble within and between groups II and III members, but that these do not assemble with group I members, which only heterodimerize within group I^30,31^. This led us to consider possible assembly of mGluR2 with mGluR7. We found that mGluR2 expression overlaps with that of mGluR7 (Supplementary Fig. 3a–c). We used anti-mGluR7 antibody to immune-precipitate mGluR7 from adult rat hippocampi and found that mGluR2 co-precipitates with mGluR7 (Supplementary Fig. 3d). To further test the potential for mGluR7 heterodimerization in native tissue, we asked whether mGluR3 and mGluR7 interact in rat cortex. We find that we mGluR3 co-precipitates with mGluR7 (Supplementary Fig. 3d). These results suggest that mGluR7 either heterodimerizes with mGluR2 in the hippocampus and with mGluR3 in the cortex, or potentially associates with these proteins indirectly.

### mGluR2/7 has high glutamate affinity

**Fig. 3:**
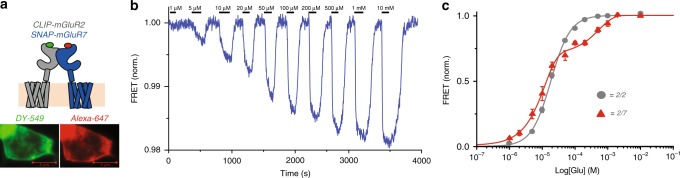
mGluR2/7 heterodimer has a biphasic glutamate concentration-response in HEK293 cells. **a** mGluR2/7 heterodimer formed by CLIP-mGluR2 and SNAP-mGluR7 heterodimer. Schematic (top) and images of HEK293 cells (bottom, scale bars 5 µm) labeled with donor (BC-549) on CLIP-mGluR2 and acceptor (BG-Alexa-647) on SNAP-mGluR7. HEK293 cells expressing SNAP-mGluR7 and CLIP-mGluR2. **b** Representative live-cell FRET trace of CLIP-mGluR2/SNAP-mGluR7 heterodimers in response to ascending concentrations of glutamate. **c** Glutamate concentration-response relation of normalized FRET in CLIP-mGluR2/SNAP-mGluR7 heterodimer (red, *n* = 3, s.e.m. error bars) compared to SNAP-mGluR2/2 (gray, *n* = 3, s.e.m. error bars).

Having found that mGluR7 associates with mGluR2, we examined the functional properties of the mGluR2/7 heterodimer. We co-expressed SNAP-mGluR7 and CLIP-mGluR2 in HEK 293 cells and labeled SNAP-mGluR7 with the SNAP-selective acceptor dye BG-Alexa 647 and CLIP-mGluR2 with the CLIP-selective donor dye BC-DY-547 (Fig. 3a). Dye labeling was specific for SNAP or CLIP and labeling of SNAP-mGluR7 and CLIP-mGluR2 was comparable (Supplementary Fig. 3f, g), indicating that they expressed at comparable levels. The co-expression of SNAP-mGluR7 and CLIP-mGluR2 is expected to yield three dimer stoichiometries: the homodimeric SNAP-mGluR7/SNAP-mGluR7; the homodimeric CLIP-mGluR2/CLIP-mGluR2, and the heterodimeric SNAP-mGluR7/CLIP-mGluR2. Of these, only the heterodimer combines the donor and acceptor and produces a FRET signal^34^.

Live-cell ensemble FRET experiments on SNAP-mGluR7/CLIP-mGluR2 revealed large glutamate-induced concentration-dependent decreases in FRET for mGluR2/7 (Fig. 3b). The behavior of mGluR2/7 differed greatly from that of the mGluR7/7. mGluR2/7 had responses to low micromolar glutamate, whereas mGluR7/7 required 10 mM glutamate to give a measurable response (compare Fig. 3b to Fig. 1b). The reverse protein tagging—CLIP-mGluR7/SNAP-mGluR2—showed a similar high apparent affinity for glutamate (Supplementary Fig. 3h). The glutamate concentration-response relation of mGluR2/7 was biphasic, consisting of a major component (~80% of total ΔFRET) with high affinity (EC50_high_ = 2.7 μM SEM: 3.8 µM), which is 7 fold lower than mGluR2/2, and a minor component (~20% of total ΔFRET) with lower affinity (EC50_low_ = 2.5 mM SEM: 0.58 mM) (Fig. 3c).

**Fig. 4:**
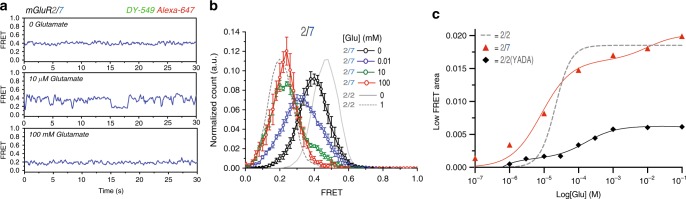
The mGluR2/7 heterodimer has high glutamate affinity and high efficacy. **a** Representative smFRET traces of CLIP-mGluR2/SNAP-mGluR7 labeled with BC-549 (donor) and BG-Alexa-647 (acceptor), in different glutamate concentrations, imaged at 10 Hz. **b** smFRET histograms of CLIP-mGluR2/SNAP-mGluR7 labeled with BC-549 and BG-Alexa-647 at 0 (5 movies, 191 molecules, s.e.m. error bars), 10 μM (7 movies, 267 molecules, s.e.m. error bars), 10 mM (6 movies, 167 molecules, s.e.m. error bars), and 100 mM (4 movies, 120 molecules, s.e.m. error bars) glutamate concentrations. Gray traces represent histograms of SNAP-mGluR2/SNAP-mGluR2 in 0 and 1 mM glutamate (solid and broken lines, respectively). **c** Glutamate concentration-response relations of low FRET (activated state) peak of smFRET histograms of SNAP-mGluR2/SNAP-mGluR2 (gray dashed line single Boltzmann fit), CLIP-mGluR2/SNAP-mGluR7 (red triangles and red solid double Boltzmann fit) and CLIP-mGluR2/SNAP-mGluR2(YADA) (black diamonds and black solid double Boltzmann fit).

Live cell assays face challenges at both low and high glutamate concentrations. Unhealthy cells may release glutamate and interfere with detection of responses to low micromolar glutamate, and it is not possible to go above 10 mM glutamate. We therefore turned to single-molecule analysis on the purified protein, where glutamate concentration could be accurately defined throughout the concentration range and where we measure absolute FRET. Individual smFRET trajectories showed that in zero glutamate mGluR2/7 heterodimers spend most of the time at a FRET level of ~0.4, whereas at 10 mM glutamate they were mainly at a low FRET level of ~0.2, and at 10 µM glutamate they transitioned back and forth between these levels (Fig. 4a). Occupancy histograms that pooled many mGluR2/7 dimers showed ~50% occupancy of the low FRET active state at 10 µM glutamate (Fig. 4b, c). At 10 mM glutamate, mGluR2/7 mostly occupied the low FRET activated state, but there remained a small high FRET shoulder, which disappeared at 100 mM glutamate (Fig. 4b). The low smFRET (activated state) concentration-response relation for mGluR2/7 was biphasic, consisting of a major (~80%) high affinity component (EC50_high_ = 8.4 μM) and a minor (~20%) low affinity component (EC50_low_ = 9.9 mM) (Fig. 4c).

We wondered if the glutamate response of mGluR3/7 heterodimers would also differ from that of the respective homodimers. Samples were prepared in the same manner as for mGluR2/7 heterodimers and imaged in smFRET. We found that, in absence of glutamate, mGluR3/7 occupies the active state ~70% of the time (Supplementary Fig. 4a), significantly higher than what we previously observed in the mGluR3/3 homodimer^13^. In 10 μM glutamate the active state occupancy was ~95% (Supplementary Fig. 4a).

We wondered whether the distinct components of the FRET concentration-response in mGluR2/7 reflect the closure at the different LBDs, i.e., if the high affinity mGluR2 LBD closes in sub-100 µM range and the low affinity mGluR7 LDB closes in the above-100 µM range. To examine this, we tested the behavior of a heterodimer that co-assembles a wild-type mGluR2 with a binding site mutant version of mGluR2, mGluR2 (Y216A and D295A; known as the YADA mutant). This YADA mutant was developed by J.P. Pin’s group in mGluR5, where, as a homodimer, it was shown to not be activated by 10 mM glutamate and was shown to have a biphasic effector activation concentration-response as a heterodimer with a wild-type subunit of mGluR5 [mGluR5/mGluR5(YADA)]^46^. The biphasic concentration-response was attributed to binding glutamate at low concentration by the wild-type LBD and at higher concentration by the YADA LBD, and closure of a single LBD was interpreted to be sufficient for only partial activation. We co-expressed CLIP-mGluR2 and SNAP-mGluR2(YADA) and labeled with the SNAP-selective acceptor dye BG-Alexa-647 and the CLIP-selective donor dye BC-DY-547. The mGluR2/mGluR2(YADA) dimer had a biphasic FRET concentration-response relation (Fig. 4c), confirming earlier observations^13^, and supporting the notion that the biphasic concentration-response relation of mGluR2/7 does indeed represent the closure over different concentration ranges of the high affinity mGluR2 LBD and the low affinity mGluR7 LBD. The high affinity component of mGluR2/mGluR2(YADA) produced only a small fraction (~10%) of maximal activation, and this stood in stark contrast to what we observed in mGluR2/7 where the first component was 10-fold larger (Fig. 4c). These findings indicate that closure of a single wild-type mGluR2 subunit is much more efficient in activating the receptor when partnered in the dimer with an mGluR7 than with another mGluR2.

### Enhanced efficacy of one-subunit liganding in mGluR2/7

The above results suggest that the heterodimeric interaction between mGluR2 and mGluR7 more strongly favors the activated state than do homodimeric interactions. We explored this further by examining trans-activation, whereby binding of ligand to one subunit activates G-protein interaction in the partner subunit^46,47^. To specifically activate only the mGluR2 subunit of the mGluR2/7 heterodimer, we selectively attached a photoswitched tethered ligand to mGluR2. We used the photoswitchable tethered glutamate, benzylguanine azobenzene glutamate (BGAG_12_), which attaches covalently to the N-terminal SNAP tag of SNAP-mGluR2, where we conjugate fluorophore for our FRET analysis. As shown earlier^48^, this BGAG_12_-labeled SNAP-mGluR2 is photo-activated by illumination at 380 nm. The tether of BGAG_12_ is short, so it only ligands and activates the subunit to which it is attached^31^. To cripple G protein signaling of the photoswitch-controlled SNAP-mGluR2 subunit, we introduced the mutation F756D, which prevents G-protein coupling^49^. SNAP-mGluR2(F756D) was co-expressed with wild-type mGluR7 along with the GIRK1 channel. The co-expression was expected to yield three mGluR dimer stoichiometries: the homodimeric SNAP-mGluR2(F756D)/SNAP-mGluR2(F756D), where both subunits would be conjugated to BGAG_12_ but neither could couple to G protein; the homodimeric mGluR7/mGluR7, which would be non-responsive to light because of lack of an attached BGAG_12_; and the heterodimeric SNAP-mGluR2(F756D)/mGluR7, the only dimer potentially capable of signaling if BGAG_12_ activation of the signaling-dead mGluR2 were able to trans-activate mGluR7 (Supplementary Fig. 4b).

Activation of BGAG_12_ on the SNAP-mGluR2(F756D) subunit by illumination at 380 nm induced an inward GIRK1 current that was reversed by illumination at 500 nm, the wavelength that causes unbinding (Supplementary Fig. 4c, d). As expected, cells expressing only the G-protein-coupling-dead SNAP-mGluR2(F756D) had no photocurrent (Supplementary Fig. 4d). These results demonstrate that the mGluR2/7 heterodimer possesses trans-activation. This result was confirmed by activation of GIRK1 channels with free glutamate over a concentration range corresponding to the high affinity activation rearrangement phase in mGluR2/7 (Supplementary Fig. 4e). As seen in the smFRET measure of the conformational change associated with activation, the foot of the curve was shifted to the left compared to that of the mGluR2/2 homodimer. The amplitude of the photocurrent was 19.7 + 1.5 % (*n* = 42 cells) that of the current induced by 1 mM glutamate (Supplementary Fig. 4b).

**Fig. 5:**
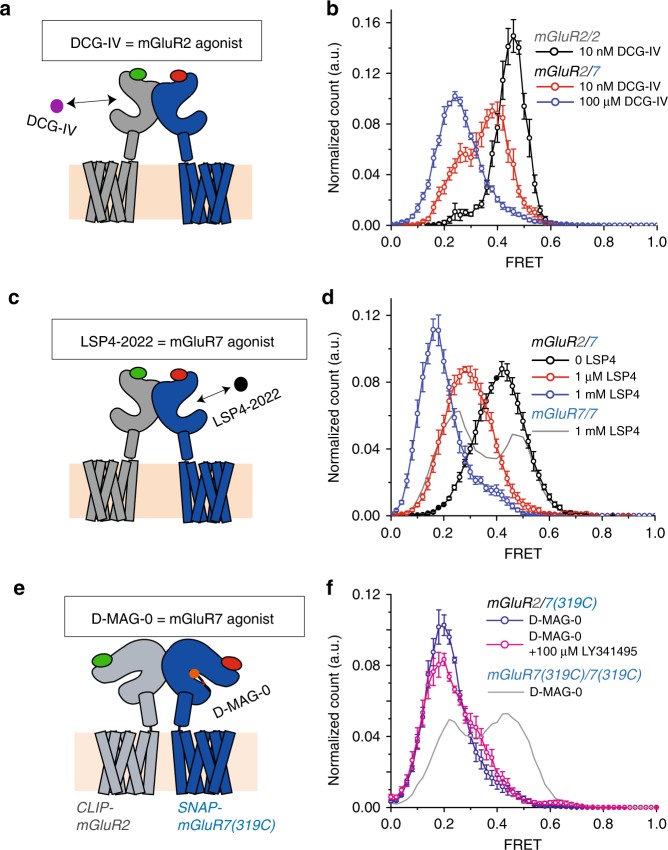
Enhanced agonism in the mGluR2/7 heterodimer. Single subunit agonism in mGluR2/7 drives greater activated state occupancy (lower FRET values) than agonism of both subunits in either mGluR2/2 or mGluR7/7. **a** Schematic of CLIP-mGluR2/SNAP-mGluR7 binds DCG-IV only in mGluR2. **b** smFRET histograms of CLIP-mGluR2/SNAP-mGluR7 and SNAP-mGluR2/SNAP-mGluR2 show greater activation at 10 nM DCG-IV in mGluR2/7 (red) (6 movies, 189 molecules, s.e.m. error bars) than in mGluR2/2 (black) (4 movies, 119 molecules, s.e.m. error bars). mGluR2/2 in the presence of 100 μM DCG-IV (blue) (4 movies, 234 molecules, s.e.m. error bars). (**c**) Schematic of CLIP-mGluR2/SNAP-mGluR7 binds LSP4-2022 only in mGluR7. **d** smFRET histograms of CLIP-mGluR2/SNAP-mGluR7 and SNAP-mGluR7/SNAP-mGluR7 show greater activation at 1 mM LSP4-2022 in mGluR2/7 (red) than mGluR7/7 (gray). CLIP-mGluR2/SNAP-mGluR7 in the presence of 0 (6 movies, 187 molecules, s.e.m. error bars), 1 μM (6 movies, 175 molecules, s.e.m error bars) and 1 mM (6 movies, 189 molecules, s.e.m. error bars) LSP4. **e** Schematic of CLIP-mGluR2/SNAP-mGluR7(K319C) with D-MAG-0 attached to K319C docking glutamate into the mGluR7 LBD in the *trans* configuration. **f** smFRET histograms of CLIP-mGluR2/SNAP-mGluR7(K319C) and SNAP-mGluR7(K319C)/SNAP-mGluR7(K319C), with *trans* D-MAG-0 (6 movies, 158 molecules, s.e.m error bars), show greater activation in mGluR2/7 (blue) than mGluR7/7 (gray). 100 µM of the orthosteric antagonist LY341495 has little effect on activation by D-MAG-0 on mGluR2/7 (magenta) (6 movies, 174 molecules, s.e.m. error bars), in contrast to the complete block seen in mGluR7/7 (Fig. 2c). **b**, **d**, **f** BC-DY-547 (donor) and BG-Alexa-647 (acceptor) imaged at 10 Hz.

We wondered if the enhanced efficacy in mGluR2/7 could be detected using a free agonist that could only activate mGluR2 and antagonize mGluR7. The concentration-response relation for effector trans-activation by the group II agonist DCG-IV measured in patch clamp recordings in cells co-expressing the GIRK1 channel showed similar concentration-response relations for mGluR7/SNAP-mGluR2(F756D) and SNAP-mGluR2/SNAP-mGluR2, but, once again, at the foot of the concentration-response relation, the mGluR2/7 heterodimer was more sensitive than the mGluR2/2 homodimer (Supplementary Fig. 4f), even though mGluR2/2 possesses two subunits that bind DCG-IV and two subunits capable of G-protein activation, whereas the heterodimer has only one. The observation of higher apparent affinity of mGluR2/7 in effector activation by DCG-IV led us to ask whether we could detect a greater potency of DCG-IV in inducing transition to the activated conformation in mGluR2/7. In smFRET, 100 μM DCG-IV induced near-complete (~90%) occupancy of the activated conformation (Fig. 5b), confirming that mGluR2/7 can be strongly activated by liganding only the mGluR2 LBD and antagonizing mGluR7^50^. At 10 nM DCG-IV, mGluR2/2 had low (~5%) occupancy of the activated conformation, but mGluR2/7 had about 5-fold greater (~25%) activated conformation occupancy (Fig. 5b). Together, these observations of activation rearrangement in the receptor and activation of the effector show that liganding of an mGluR2 LBD activates the receptor more potently when mGluR7 is the partner subunit than when the partner is another mGluR2 subunit.

Having seen that liganding of just the mGluR2 subunit can strongly activate mGluR2/7, we examined the effect of agonist binding to only the mGluR7 subunit. We isolated receptor protein from cells co-expressing SNAP-mGluR7 and CLIP-mGluR2, where the group III selective agonist LSP4-2022 could be used to bind only to the mGluR7 subunit of mGluR2/7 (Fig. 5c). We observed two striking differences between LSP4-2022 activation of the mGluR7/7 homodimer and the mGluR2/7 heterodimer. A first difference was that, whereas 1 mM LSP4-2022 produced slightly more than 50% occupancy of the activated conformation in mGluR7/7, activation was almost complete in mGluR2/7 (Fig. 5d). Indeed, even 1 µM LSP4-2022 was sufficient to shift the smFRET distribution slightly more than half-way toward the activated low FRET state (Fig. 5d). This gives the mGluR7 subunit an approximately 1000-fold higher apparent affinity for LSP4-2022 when partnered with mGluR2 than when partnered with another mGluR7. A second difference was that in mGluR2/7 LSP4-2022 concentrations near the EC50 produced a single broad distribution with a peak, which was intermediate between the high FRET resting and low FRET activated states, as opposed to two resolved distributions of the high FRET resting and low FRET activated states, as seen in mGluR7/7 (Fig. 5d). This single broad smFRET distribution is similar to what we have seen with glutamate activation of the mGluR2/7 heterodimer, above (Fig. 4b). We examine this further below.

Having seen that synthetic agonists that are selective for either the mGluR2 subunit or the mGluR7 subunit produce stronger activation in mGluR2/7 than in either mGluR2/2 or mGluR7/7, we wanted to determine if the native agonist glutamate is also a stronger activator in mGluR2/7. To confine the glutamate to one of the subunits, we once again tethered the photoswitchable tethered glutamate, D-MAG-0, to the mGluR7 LBD (Fig. 5e). smFRET analysis showed that mGluR2/7 with a tethered ligand only on the one mGluR7 subunit spends ~90% of the time in the activated low-FRET state of ~0.2, higher than the ~50% activation seen in the mGluR7/7 homodimer in which both subunits had the tethered agonist (Fig. 5f). In addition, we found that, whereas 100 µM of the competitive antagonist LY341495 almost completely blocked activation of mGluR7/7 by *trans* D-MAG-0 (Fig. 2c), it had little effect on activation by *trans* D-MAG-0 of mGluR2/7 (Fig. 5f). The results suggest that D-MAG-0 binds with higher affinity to the mGluR7 LBD when it is partnered with mGluR2 than when it is partnered with another mGluR7.

Together these experiments show that mGluR7 enhances activation by mGluR2 and mGluR2 enhances activation by mGluR7, so that the heterodimer has greater sensitivity to glutamate and synthetic agonists and stronger activation by single subunit agonism than seen in either the mGluR2/2 homodimer, or the mGluR7/7 homodimer.

### Spontaneous rearrangements and altered kinetics in mGluR2/7

One striking property of the mGluR2/7 heterodimer is the shape of the smFRET histogram at intermediate concentrations. mGluR7/7 had two well-resolved smFRET distributions—the resting high-FRET state and the activated low-FRET state (Figs. 1e and 2)—and the occupancy of the former decreases as occupancy of the latter increases with a progressive rise in agonist concentration, as also observed earlier in the mGluR2/2 and mGluR3/3 homodimers and the mGluR2/3 heterodimer^13,31^. In contrast, the mGluR2/7 heterodimer smFRET histogram is a single, broadened distribution, which shifts progressively to lower FRET levels as agonist concentration increased, whether the agonist was glutamate, which could bind to both the mGluR2 and mBGluR7 LBDs (Fig. 4b), or the group III-selective ligand LSP4-2022, which binds to only the mGluR7 LBD (Fig. 5d).

**Fig. 6:**
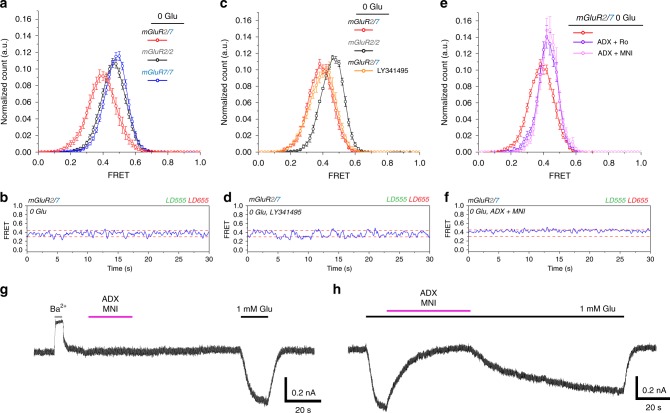
Spontaneous conformational rearrangements of mGluR2/7 in the Apo state. **a**, **b** 0 glutamate smFRET histograms of SNAP-mGluR2/SNAP-mGluR2, SNAP-mGluR7/SNAP-mGluR7 and CLIP-mGluR2/SNAP-mGluR7 show a shift to lower FRET values in mGluR2/7 (**a**), associated with spontaneous transitions to an intermediate FRET state of ~0.3. **c**, **d** The left shift of the 0 glutamate CLIP-mGluR2/SNAP-mGluR7 (5 movies, 191 molecules, s.e.m. error bars) smFRET histogram relative to that of SNAP-mGluR2/SNAP-mGluR2 (6 movies, 257 molecules, s.e.m. error bars) (**c**) and the spontaneous transitions to the intermediate FRET state (**d**) are not altered by 100 µM of the orthosteric antagonist LY341495 in 0 glutamate (5 movies, 230 molecules, s.e.m. error bars). **e**, **f** The left shift of the 0 glutamate CLIP-mGluR2/SNAP-mGluR7 smFRET histogram (**e**) and the spontaneous transitions to the intermediate FRET state (**f**) are blocked by combined negative allosteric modulators for mGluR2 (either 11 µM Ro-5229 (6 movies, 160 molecules, s.e.m. error bars) or 1.2 µM MNI-137 (6 movies, 154 molecules, s.e.m. error bars)) and mGluR7 (125 µM ADX 71743) in 0 glutamate. **a**–**f** BC-DY-547 or BG-DY-547 (donor) and BG-Alexa-647 (acceptor) imaged at 10 Hz. **g**, **h** Whole cell patch clamp recording (V_h_ = −60 mV; [K^+^]_in_ = [K^+^]_out_ = 150 mM) in HEK293T cells co-expressing mGluR2, mGluR7 and the GIRK1(F137S) channel have basal inward current, which is reversibly inhibited by the GIRK pore blocker Ba^2+^, but not affected by the combined negative allosteric modulators for mGluR2 (1 µM MNI-137) and mGluR7 (3 µM ADX 71743) (**g**), even though these are sufficient to completely block activation by 1 mM glutamate (**h**).

We examined this further and found that at zero glutamate the smFRET distribution of mGluR2/7 was also broadened and left-shifted with respect to both mGluR2/2 and mGluR7/7 at zero glutamate (Fig. 6a). One possible explanation for such behavior is spontaneous closure of LBDs leading to basal activation. To test this, we applied 100 µM of the group II and group III competitive antagonist LY341495. LY341495 binds in the orthosteric site, holds the LBD in the open conformation^30^ and blocks the activation rearrangement that is induced by glutamate bound in the orthosteric site of mGluR2/2 and mGluR3/3, as well as by calcium ion binding at an allosteric site in mGluR3/3^13^ (PDB 3MQ4). However, LY341495 did not alter the smFRET distribution of the mGluR2/7 heterodimer (Fig. 6c), indicating that the broadening and shift to the left in mGluR2/7 is not due to spontaneous LBD closure in the Apo state.

mGluRs respond to agonist in a sequence of steps: agonist binding favors closure of the LBDs, the closed LBDs rotate, and this triggers a rotation of the transmembrane domain (TMD) that opens the intracellular-facing G protein docking site^29,51^. We tested negative allosteric modulators (NAMs) of mGluR7 (ADX*-*71743) and mGluR2 (Ro 64*-*5229 and MNI-137), which bind in the TMD and stabilize the resting state^52–55^ and, so, would be expected to stabilize the open and non-rotated (resting) conformation of the LBD. A combination of either of the mGluR2 NAMs with the mGluR7 NAM narrowed the smFRET distribution and shifted it to the right (Fig. 6e), as would be expected for a negative allosteric effect that pushes a partially activated receptor into the resting state.

To understand the molecular motions underlying the shift in smFRET distribution, we analyzed single molecule trajectories in zero glutamate. We observed spontaneous transitions from a high FRET resting state of ~0.45 to an intermediate FRET state of ~0.3 (Fig. 6b and Supplementary Fig. 5, top). These spontaneous transitions persisted in the presence of LY341495 (Fig. 6d and Supplementary Fig. 5, second from top), but were eliminated by the NAMs, which stabilized the receptors in the high FRET state (Fig. 6f, and Supplementary Fig. 5, bottom two). These findings suggest that, in the Apo state, mGluR2/7 spontaneously transitions into a conformational intermediate on the activation pathway in which the LBDs are open but rotated.

We next asked whether the Apo state intermediate of mGluR2/7 represents the G protein signaling state of the receptor. To this test this we co-expressed mGluR2 and mGluR7 in HEK293T cells along with the GIRK channel and measured GIRK current. Application of 1 mM barium, to block the GIRK channels, elicited a reduction in standing inward current, which could be due to either or both basal activity of the receptor and excess free Gβγ (Fig. 6g). 1 mM glutamate elicited a large inward current above this basal level and this was completely blocked (96.7 ± 2.1%, *n* = 4) by a combination of 3 μM ADX71743 (the mGluR7 NAM) and 1 μM MNI-137 (the mGluR2 NAM) (Fig. 6g, h). However, the combined NAMs on their own had no effect on basal current (2.1 ± 7.3%, *n* = 6, *t*-test *p* = 0.542). These results suggest that the intermediate conformation visited in the Apo state is associated with a non-signaling state of the TMD.

**Fig. 7:**
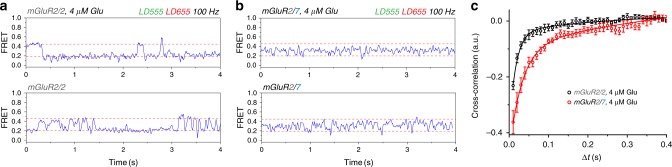
Fast glutamate-induced conformational dynamics in mGluR2/7. SNAP-mGluR2/SNAP-mGluR2 (mGluR2/2) and CLIP-mGluR2/SNAP-mGluR7 (m,GluR2/7) at a glutamate concentration near the EC50 (4 µM) imaged at 10× speed (100 Hz) with photobleaching resistant dyes (donor BC-LD555 or BG-LD555 and acceptor BG-LD655). **a**, **b** mGluR2/2 and mGluR2/7 transition between resting (high FRET) and activated (low FRET), but CLIP-mGluR2/SNAP-mGluR7 spends much of the time at an intermediate FRET level. **c** Donor/acceptor cross-correlation decays over longer time in mGluR2/7. Both samples fit with double exponential decay, mGluR2/2 R^2^ = 0.9120 (8 movies, 396 molecules, s.e.m. error bars) and mGluR2/7 R^2^ = 0.9740 (8 movies, 384 molecules, s.e.m. error bars).

An intermediate FRET state of ~0.3 has been observed previously in mGluR2/2, but it was very short-lived and could only be visualized as a low-incidence shoulder when large numbers of transitions, acquired at a glutamate concentration near the EC50, were aligned temporally^13^. We examined the single-molecule activation-deactivation dynamics at near the glutamate EC50 for mGluR2/7. We performed these experiments with a more photo-stable donor/acceptor dye pair (LD555/LD655, Förster radius 52 Å) and 10-times faster acquisition (100 Hz). We found that, unlike mGluR2/2, which toggles back and forth between the resting 0.45 and activated 0.20 FRET states, with rarely resolved, brief occupancies at intermediate FRET levels (Fig. 7a and Supplementary Fig. 6a), mGluR2/7 divides its time between the ~0.45 FRET state, the ~0.20 FRET state and a ~0.30 intermediate FRET level, with short-lived occupancies of the resting and activated conformations and substantial time spent in the intermediate conformation (Fig. 7b and Supplementary Fig. 6b). The distinct activation/deactivation kinetics are reflected in a more extended donor/acceptor cross-correlation in mGluR2/7 (Fig. 7c).

Thus, along with enhanced affinity and boosted single subunit activation, the mGluR2/7 heterodimer displays a unique form of spontaneous partial activation, in absence of agonist and with the LBDs open, as well as altered conformational dynamics.

## Discussion

The mGluR7/7 homodimer is known, based on effector activation, to have unusually low apparent affinity for glutamate^23^. Our measure of the glutamate-induced structural rearrangement of the LBDs reveals an EC50 of 38 mM, ~4000-fold lower than that of the other mGluRs. Along with this, we find that maximal activation by glutamate only reaches ~10%, as determined from occupancy of the activated conformation, where the LBD clamshells are both closed and rotated, producing our measured low-FRET state. Despite this large difference in relative occupancy, the absolute smFRET levels of the resting and activated states in mGluR7/7 are similar to those of mGluR2/2, mGluR3/3, and mGluR2/3^13,31^, suggesting that mGluR7/7 undergoes the same activation rearrangements. We show that occupancy of the activated conformation is elevated by a mutation that increases glutamate affinity, or by presentation of the glutamate on a stiff tethered linker that docks it into the binding pocket, or by a group III selective synthetic agonist, indicating that the problem with mGluR7/7 is not is ability to activate, but the ability of free glutamate to stabilize the activated conformation.

The very low affinity and low efficacy of activation by glutamate of mGluR7/7 poses a functional conundrum. Even within the low volume synaptic cleft, and immediately after glutamate release, glutamate is estimated to reach a peak of only 1–3 mM peak and for less than one millisecond^37,38^. Spillover from nearby synapses is thought to reach much lower concentrations^56^. Thus, the affinity and efficacy of mGluR7/7 appear to be too low for significant activation under physiological conditions. And yet, pharmacological and genetic knock-out studies suggest that mGluR7 does function at synapses^57–59^. Also, the pharmacological profile differs from what is measured from mGluR7 expressed alone in non-neuronal cell lines, and that which is measured in neuronal tissue^60^ suggesting a biochemical difference between mGluR7 in neurons and in non-neuronal cells. We considered that heterodimerization, which is known to occur between mGluR7 and other group III mGluRs, as well as with group II mGluRs, may yield a receptor that operates in the range of physiological glutamate. We found that mGluR7 expression overlaps with that of mGluR2 in the hippocampus, that mGluR7 and mGluR2 co-immune precipitate. Strikingly, the mGluR2/7 heterodimer has high affinity and efficacy. But heterodimerization with mGluR2 does more than simply bring mGluR7 into the physiological range, it activates more efficiently and at lower agonist concentration than even mGluR2/2. This is associated with a form of cooperativity, in which an unliganded mGluR2 greatly enhances activation by agonist bound to mGluR7, and *vice versa*, and with a unique conformational pathway to activation, in which mGluR2/7 partially activates in the Apo state, even when its LBD is held open by antagonist.

mGluRs must dimerize to signal^32,61,62^, and, in the dimer, activation of only one subunit produces low efficacy activation. This was originally shown in mGluR5/5, where a wild-type subunit was dimerized with one carrying the YADA binding site mutant subunit, which drastically lowers affinity^46^. The glutamate concentration-response curve of mGluR5/mGluR5(YADA) is biphasic: a first low glutamate concentration component, where only the wild-type subunit is expected to bind, yields ~20% of maximal activation achieved at much higher glutamate concentration, which is expected to also bind the YADA subunit. Similar behavior has been described for mGluR2/mGluR2(YADA)^13^ and we confirm that here. We find that maximal activation of mGluR2/mGluR2(YADA)—at high glutamate (where both the high affinity wild-type and low affinity YADA subunits are both expected to be glutamate bound—only yields ~30% occupancy of the low FRET activated conformation, in contrast to the full occupancy seen in the wild-type mGluR2/2 dimer.

mGluR2/7 is expected to function similarly to mGluR2/mGluR2(YADA), with the naturally very low affinity wild-type mGluR7 subunit taking the place of the mutant mGluR2(YADA) subunit. However, mGluR2/7 breaks expectation in two ways. First, compared to mGluR2/2, the mGluR2/7 heterodimer activates at lower glutamate concentrations. Second, in contrast to mGluR2/mGluR2(YADA), mGluR2/7 has ~4-fold higher efficacy at 1 mM glutamate, the top of the first component of the biphasic concentration-response relation, when only the wild-type mGluR2 subunit is expected to be liganded (~80% of maximal activation) and achieves full occupancy of the low FRET activated conformation at the top of the biphasic concentration-response relation, ~3-fold better than the maximal activation of mGluR2/mGluR2(YADA).

Compared to mGluR7/7, the mGluR2/7 heterodimer is activated with higher efficacy by both the group III selective agonist LSP4-2022 and by the tethered glutamate photoswitch D-MAG-0, even though in mGluR7/7 both subunits are bound by agonist, whereas in the mGluR2/7 heterodimer only the mGluR7 subunit has the agonist and the mGluR2 subunit is in the Apo state. This suggests that agonist-induced closure of the mGluR7 LBD drives the empty mGluR2 LBD to also close and rotate. This cooperative effect works in the opposite direction also: The presence of the mGluR2 subunit enhances agonist binding by the mGluR7 subunit. This is seen with the competitive antagonist 100 μM LY341495, which appears to completely displace D-MAG-0 from the binding site in the mGluR7/7 homodimer (shifting completely to the resting high FRET state), whereas in the mGluR2/7 heterodimer 100 μM LY341495 has almost no effect, suggesting that D-MAG-0 has a much higher affinity for mGluR7 when it is paired in the heterodimer with mGluR2. Thus, mGluR2/7 has a unique form of heteromeric cooperativity in which each subunit in its Apo state acts as a positive allosteric modulator of its partner subunit.

While some GPCRs, such as rhodopsin, have very low basal activation, others activate in absence of agonist^63^. Basal activation in absence of glutamate occurs in some mGluRs, including in mGluR3/3 and mGluR2/3, which are partially activated by calcium binding near the mGluR3 orthosteric site^13,64,65^. We see fundamental differences between basal activation of mGluR2/7 and examples described previously. mGluR3/3 and mGluR2/3 basal activation appears as FRET transitions from ~0.45 FRET to ~0.2 FRET and this transition is mediated by LBD closure and can be blocked by orthosteric antagonist LY341495 which stabilizes the open conformation of the LBD. However, the basal activity in mGluR2/7 is characterized by transitions between ~0.45 FRET and intermediate ~0.3 FRET and is not affected by LY341495, but is blocked by NAMs, which stabilize the inactive conformation of the TMD.

**Fig. 8:**
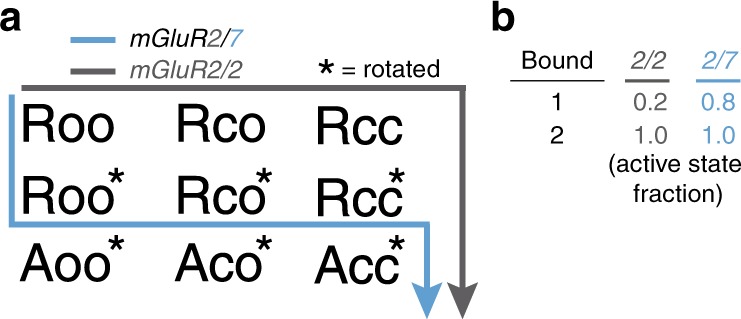
State diagram model of activation pathway. **a** The transition of an mGluR from the resting state (Roo) to the active state (Acc) involves transition of the two LBDs from the open state (o) to the closed state (o), and rotation (*) of the LBDs relative to one another. In absence of agonist, mGluR2/2 is stable in Roo, and previous work has shown that the main route of activation is Roo → Roc → Rcc → Rcc*, although a small amount of activation is possible from Roc. In contrast, we find that mGluR2/7 spontaneously enters a partially activated (functionally inactive) conformation when both LBDs are held open by antagonist, which we propose as Roo → Roo*. **b** The energetic difference results in much higher occupancy of the fully active state in mGluR2/7 in which only one subunit is liganded by agonist.

Activation is thought to proceed from the resting state (R) to the active state (A), starting with closure of the LBDs on the agonist (Roo → Rcc), followed by LBD rotation (Rcc → Rcc*), and by stabilization of a rearranged state of the TMD that includes a whole-TMD rotation and a tilt of TM6 to open the G-protein binding pocket: Rcc* → Acc* (Fig. 8a)^29,66^. Since spontaneous entry into the intermediate state occurs with the LBDs held open by LY341495 in zero glutamate (so that LBD closure does not contribute to the reduced FRET), and NAMs prevent entry into this state, we propose that the intermediate FRET state reflects a partially activated conformation in which the LBD is open but rotated. Our functional analysis indicates that this partially activated conformation is signaling inactive (Roo*) (Fig. 8a). This unusual activation pathway in mGluR2/7 is associated with a unique potency of single subunit liganding, which achieves ~80% of maximal activation as compared to only ~20% seen in mGluR2/mGluR2(YADA) (Fig. 8b).

Near the EC50 glutamate concentration, where the receptor spends roughly equal time in the resting and activated states, mGluR2/7 is more dynamic than mGluR2/2 and spends substantial time in the partially activated state. Thus, the enhanced affinity and efficacy of mGluR2/7 are associated with a favored transition to a partially activated state where the LBD is rotated but the TMD is in the non-signaling state. Recent work has provided the first structure of a full length, dimeric mGluR complex in the resting and active conformations^29^ that are compatible with earlier evidence for TMD rotation^66^. Our observations indicate that the conformational pathway between these states has several possible paths.

In conclusion, our study reveals a specialized heteromeric cooperativity in mGluRs in which an unliganded subunit confers on its dimer partner both enhanced glutamate affinity and the ability of the single subunit bound state to fully activate the receptor. This cooperativity is associated with a conformational pathway to activation that is not seen in other mGluRs. Instead of the canonical pathway of sequential LBD closure on agonist followed by LBD rotation, it appears that the LBD rotates even when it is open, leading to a partially activated state even when both members of the dimer are in the Apo state. Unusually too, while threshold sensitivity is enhanced, the concentration-response relation of the mGluR2/7 heteromer is shallower overall and saturates at higher concentration, adding to the ability to respond to low concentration spillover from a distance, a wider dynamic range to respond proportionately to particularly high glutamate from nearby sources.

## Methods

### Drugs

**Fig. 9:**
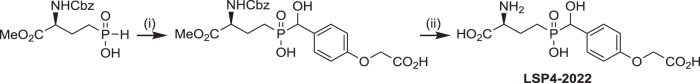
Improved synthesis of LSP4-2022^a^. ^a^Reagents and conditions: (i) 4-(Carboxymethoxy)benzaldehyde, BSA, degassed CH_2_Cl_2_, RT, 16 h; (ii) **a** 6 M aqueous HCl, reflux, 5 h; **b** C-18 flash chromatography. Abbreviations: BSA: *N,O-*bis(trimethylsilyl)acetamide; Cbz: benzyloxycarbonyl; L-AP4: L-2-amino-4-phosphonobutyric acid; Me: methyl; RT: room temperature.

LSP4-2022 was obtained according to the general procedure following Fig. 9^67^, with improvements to minimize the formation and discard L-AP4, a side product with selective and potent group III mGluR agonist activity: (i) The use of degassed solvent during the formation of the second C-P bond dramatically reduces the presence of the corresponding phosphonate; (ii) The purification of the crude LSP4-2022 by C-18 chromatography using a slow gradient (H_2_O + 0.1% HCO_2_H/MeCN + 0.1% HCO_2_H: from 100/0 to 90/10), instead of successive cation- and anion- exchange resins, saves time and increases the overall yield of the final compound.

### Cell culture and transfection

HEK293T cells were cultured in DMEM with 5% FBS on poly-L- lysine-coated glass coverslips. HEK293T cells were obtained from the UC Berkeley MCB tissue culture facility, authenticated by DDC Medical, and tested negative for mycoplasma contamination. Previously described HA–SNAP and Flag–CLIP-tagged rat mGluR cDNA were generously provided by J. P. Pin. DNA plasmids were transfected into cells using lipofectamine 2000 (Thermo Fisher). For electrophysiology experiments, cells were transfected with wild-type-mGluR2 or wild-type- mGluR7 or SNAP-mGluR2(F756D), GIRK1-F137S, and yellow fluorescent protein (YFP) (as a transfection marker) at a 7:7:1 ratio with 0.7 mg plasmid per well for receptor and channel. For FRET experiments, cells were transfected with SNAP and CLIP-tagged constructs at a ratio of 1:2 with 0.3 mg of SNAP–mGluR DNA per well.

### Patch clamp electrophysiology

Whole-cell patch clamp recordings from single isolated cells were performed 24–48 h after transfection, in a high potassium extra-cellular solution containing (in mM): 120 KCl, 29 NaCl, 1 MgCl_2_, 2 CaCl_2_ and 10 HEPES, pH 7.4. Cells were voltage clamped to −60 mV using an Axopatch 200B amplifier (Axon Instruments) and membrane currents were recorded. Glass pipettes of resistance between 3 and 8 MΩ were filled with intracellular solution containing (in mM): 140 KCl, 10 HEPES, 3 Na_2_ATP, 0.2 Na_2_GTP, 5 EGTA and 3 MgCl_2_, pH 7.4. Data were acquired with a 2 kHz acquisition rate and low-pass filtered with a 4-pole Bessel filter at 1 kHz. Data acquisition and analysis were performed using pCLAMP 10 software (Axon Instruments).

### Photoswitching

Illumination was applied to the entire field of view using either a Polychrome V monochromator (TILL Photonics) through a 20× objective, or a Lambda DG4 high-speed wavelength switcher (Sutter Instruments) with 380 nm and 500 nm filters through a 40× objective. pClamp software was used for both data acquisition and control of illumination. BGAG labeling was done by applying 1.5 μM BGAG for 45 min at 37 °C. To conjugate D-MAG-0, cells were incubated in 50–100 µM D-MAG-0for 30–60 min in the dark at 23–27 °C in standard extracellular cell buffer.

### FRET pair fluorescent dye labeling of protein-tagged mGluRs

Approximately 24–48 h after transfection, cells were labeled while attached to poly-L-lysine-coated coverslips. Culture media was removed and coverslips were washed and transferred to extracellular solution containing (in mM): 135 NaCl, 5.4 KCl, 2 CaCl_2_, 1 MgCl_2_, 10 HEPES, pH 7.4. Cells were labeled at 37 °C with one or two SNAP-reactive (benzylguanine, BG) dyes at 1.5 μM for 45 min, and then, if a CLIP-tagged mGluR was used, they were washed and labeled with a CLIP-reactive (benzylcytosine, BC) dye at 3 μM for 45 min. For most of the experiments (Figs. 1–6 and Supplemental Figs. 1–5, except Supplemental Fig. 1c) DY-547 (NEB) was used as a donor and Alexa-647 (NEB) as an acceptor (in Supplemental Fig. 1c Atto-488 was the donor and Atto-594 was the acceptor; Company). In Fig. 7 and Supplemental Fig. 6, LD-655 was the donor and LD-655 was the acceptor (Lumidyne). The fluorophores were diluted in extracellular solution and coverslips were washed in between labeling with donor and acceptor.

### Ensemble FRET

After labeling, cells were mounted on an upright, scanning confocal microscope (Zeiss LSM 780) and imaged with a 20X objective. Donor excitation was performed using a 561-nm laser and images were taken in the donor and acceptor channels at 1 Hz. Clusters of cells were analyzed together and FRET was calculated as FRET = (*I*_A_)/(*I*_D_ + *I*_A_), in which *I*_D_ is the fluorescence donor intensity and *I*_A_ is the fluorescence acceptor intensity. For individual traces, FRET was normalized to the basal FRET value observed before application of drugs. FRET changes calculated for dose–response curves were normalized to the response to saturating glutamate (10 mM) and dose–response curves were obtained from multiple cell clusters and averaged from at least three experiments. Fitting of dose–response curves was performed using Prism (Graphpad). All drugs, except LSP4-2022, which we synthesized, were purchased from Tocris. Drugs were delivered with a gravity-driven perfusion system.

### smPull receptor isolation and surface display

To inhibit nonspecific protein adsorption, flow cells for single-molecule experiments were prepared as previously described^13^ using mPEG (Laysan Bio) passivated glass coverslips (VWR) and doped with biotin PEG16. Before each experiment, coverslips were incubated with NeutrAvidin (Thermo), followed by 10 nM biotinylated secondary antibody (donkey anti-rabbit, Jackson ImmunoResearch). For receptor immunopurification, 10 nM anti-mGluR2 primary antibody (abcam, ab150387) or 10 nM anti-mGluR7 antibody (abcam, ab53705), or 15 nM anti-HA antibody (abcam, ab26228) was incubated in the chamber (Fig. 1e). Between each conjugation step, the chambers were flushed to remove free reagents. The antibody dilutions and washes were done in T50 buffer (50 mM NaCl, 10 mM Tris, pH 7.5). For single-molecule experiments, fresh cells expressing tagged mGluR constructs were labeled, as described above, and recovered from coverslips by incubating with Ca^2+^ free PBS buffer for 5–10 min followed by gentle pipetting. Cells were then pelleted by spinning at 5,000 g for 5 min and lysed in lysis buffer consisting of 150 mM NaCl, 1 mM EDTA, protease inhibitor cocktail (Thermo Scientific) and 1.2% IGEPAL (Sigma).After 1 h incubation at 4 C°, lysate was centrifuged at 16,000 × *g* for 20 min. and the supernatant was collected and kept on ice. To achieve sparse immobilization of labeled receptors on the surface, the cell lysate was diluted (ranging from 5× to 50× dilution depending on the expression and labeling efficiency) and applied to coverslips. After achieving optimum surface immobilization (~400 molecules in a 2000 μm^2^ imaging area), unbound receptors were washed out of the flow chamber and the flow cells were then washed extensively (up to 50× the cell volume).

### smFRET measurements

Receptors were imaged for smFRET in imaging buffer consisting of (in mM) 3 Trolox, 120 KCl, 29 NaCl, 2 CaCl_2_, 1 MgCl_2_, 50 HEPES, 0.04% IGEPAL and an oxygen scavenging system (0.8% dextrose, 0.8 mg ml^−1^ glucose oxidase, and 0.02 mg ml^−1^ catalase), pH 7.4. Reagents were purchased from Sigma and were all UltraPure grade (purity > 99.99%). All buffers were made in UltraPure distilled water (Invitrogen). For the experiments done in the absence of Ca^2+^, 10 mM EGTA and 1 mM EDTA were added to the imaging buffer. Catalase was diluted in T50 buffer and passed through a spin column 3× (BioRad).

Samples were imaged with a 1.65 na ×60 objective (Olympus) on a TIRF microscope with 100 ms time resolution unless stated otherwise. Lasers at 532 nm (Cobolt) and 632 nm (Melles Griot) were used for donor and acceptor excitation, respectively. FRET efficiency was calculated as (*I*_A_ − 0.1*I*_D_)/(*I*_D_ + *I*_A_), in which *I*_D_ and *I*_A_ are the donor and acceptor intensity, respectively, after back-ground subtraction. Imaging was with 100 ms acquisition time (10 Hz) with an Andor iXon 897 EMCCD camera (Figs. 1–6 and Supplemental Figs. 1–5) or with 10 ms acquisition time (100 Hz) with a Photometrics Prime 95B cMOS camera (Fig. 7 and Supplemental Fig. 6) using Lumidyne LD555 as donor and Lumidyne LD655 as acceptor (Förster radius ~52 Å). Dyes were conjugated to benzyguanine and benzylecytosine to allow for labeling of SNAP and CLIP proteins, respectively.

### smFRET data analysis

Single-molecule intensity traces showing single-donor and single-acceptor photobleaching with a stable total intensity for longer than 5 s were collected (20–30% of total molecules per imaging area). Individual traces were smoothed using a nonlinear filter^68^ with following filter parameters: window = 2, M = 2 and *P* = 15. Each experiment was performed >4 times to ensure reproducibility. smFRET histograms were compiled from >100 molecules per condition. (100 ms time resolution). Error bars in the histograms represent the standard error from >4 independent movies. To ensure that traces of different lengths contribute equally, histograms from individual traces were normalized to one before compiling. Gaussian fitting to histograms was done in Origin Pro. smFRET histograms were fit to two Gaussians, centered on high (~0.45) and low (~0.2) FRET levels for mGluR2/2 and mGluR2/7 and a single Gaussian for mGluR2/7 (center ranging from 0.2 to 0.45). For dose–response relations, the area underneath the low FRET Gaussian was used as a measure of active state occupancy.

### Immunostaining for brain sections

Mice were anaesthetized and perfused transcardially with 4% paraformaldehyde (PFA) in 0.1 M phosphate buffer (pH 7.4). The brains were removed, postfixed overnight at 4 °C and sectioned at a thickness of 50 μm. Free-floating sections were incubated in blocking solution (PBS containing 10% normal sheep serum and 0.1% Triton X-100) overnight, followed by incubation with primary antibodies(diuluted 1 to 500) for 3 days and, after extensive washing, with Alexa 488- or Alexa 647-conjugated secondary antibodies (Molecular Probes) overnight at 4 °C.

### Western blot analysis

Cells were scraped into lysis buffer (50 mM Tris- HCl, pH 7.5, and 150 mM NaCl) containing 0.5% IGEPAL and protease inhibitor mixture (Roche) and incubated on ice for 20–30 min. The supernatant was separated from cell debris by centrifugation at 16,000 × g for 10 min at 4 °C. Aliquots of lysate were heated in SDS sample buffer (containing 10% SDS and 9.3% DTT) at 95 °C for 5 min. Samples were loaded on SDS–PAGE and transferred to nitrocellulose membranes (Bio-Rad). After transfer, membranes were blocked in TBST (25 mM Tris, 150 mM NaCl, and 0.05% Tween 20) containing 5% nonfat milk at room temperature for 1 h. mGluR2 antibodies, mGluR7 and mGluR3 (Novus Biologicals, cat. # NBP2-61843) antibodies were diluted in blocking solution and incubated with the membranes at 4 °C overnight. Membranes were then washed with TBST and incubated with HRP-conjugated goat anti-rabbit IgG secondary antibody (Santa Cruz Biotechnology, catalog #sc-2004, 1:7500 diluted in blocking buffer or Jackson ImmunoResearch Laboratories, catalog #111-035-144, 1:10,000 diluted in blocking buffer).

### Co-immunoprecipitation from brain tissue

Brain samples were collected from Spraque Dawely rats, suspended in lysis buffer (50 mM Tris- HCl, pH 7.5, and 150 mM NaCl) containing protease inhibitor mixture (Roche). Tissue samples were homogenized and sonicated. Cell membrane fractions were separated by centrifugation at 100,000 × *g* and cell pellet was resuspended in lysis buffer containing 1.5% IGEPAL and protease inhibitor mixture (Roche). Cell were rocked at 4 °C for one hour then supernatant was cleared by centrifugation at 16,000 × *g* for 20 min at 4 °C. Supernatant precleared with Pierce Protein A/G magnetic beads (Thermo-Fisher) followed by incubation with primary antibody conjugated protein A/G magnetic beads overnight at 4 °C. Beads were washed and protein was eluted in SDS sample buffer (containing 10% SDS and 9.3% DTT) at 95 °C for 5 min. Samples were run on western blot as described above.

## Supplementary information

Supplementary Information

## Data Availability

Data supporting the findings of this manuscript are available from the corresponding author upon reasonable request. A reporting summary for this Article is available as a Supplementary Information file. The source data underlying Figs. 1a–c, e–f, 2a–c, 3a, c, 4b–c, 5b, d, f, 6a, c, e, 7c and Supplementary Figs. 1a, d, f, h, 2a, e, 3a–g, 4a, d, e–f are provided as a Source Data file.

## Source data

Source Data
