# Supplementary material for: Conformational pathway provides unique sensitivity to a synaptic mGluR
Source: Nat Commun. 2019 Dec 5;10:5572. doi: 10.1038/s41467-019-13407-8 (PMC6895203; doi:10.1038/s41467-019-13407-8)
Supplement: Supplementary file 1 — Supplementary Information [file 41467_2019_13407_MOESM1_ESM.pdf]

Supplemental Information for:

Conformational pathway provides unique sensitivity to a synaptic mGluR

Chris Habrian[1], Joshua Levitz[2,3], Vojtech Vyklicky[2], Zhu Fu[2], Adam Hoagland[2], Isabelle McCort-Tranchepain[4], Francine Acher[4] and Ehud Y. Isacoff [1,2,5,6,] *

Supplemental Figure 1

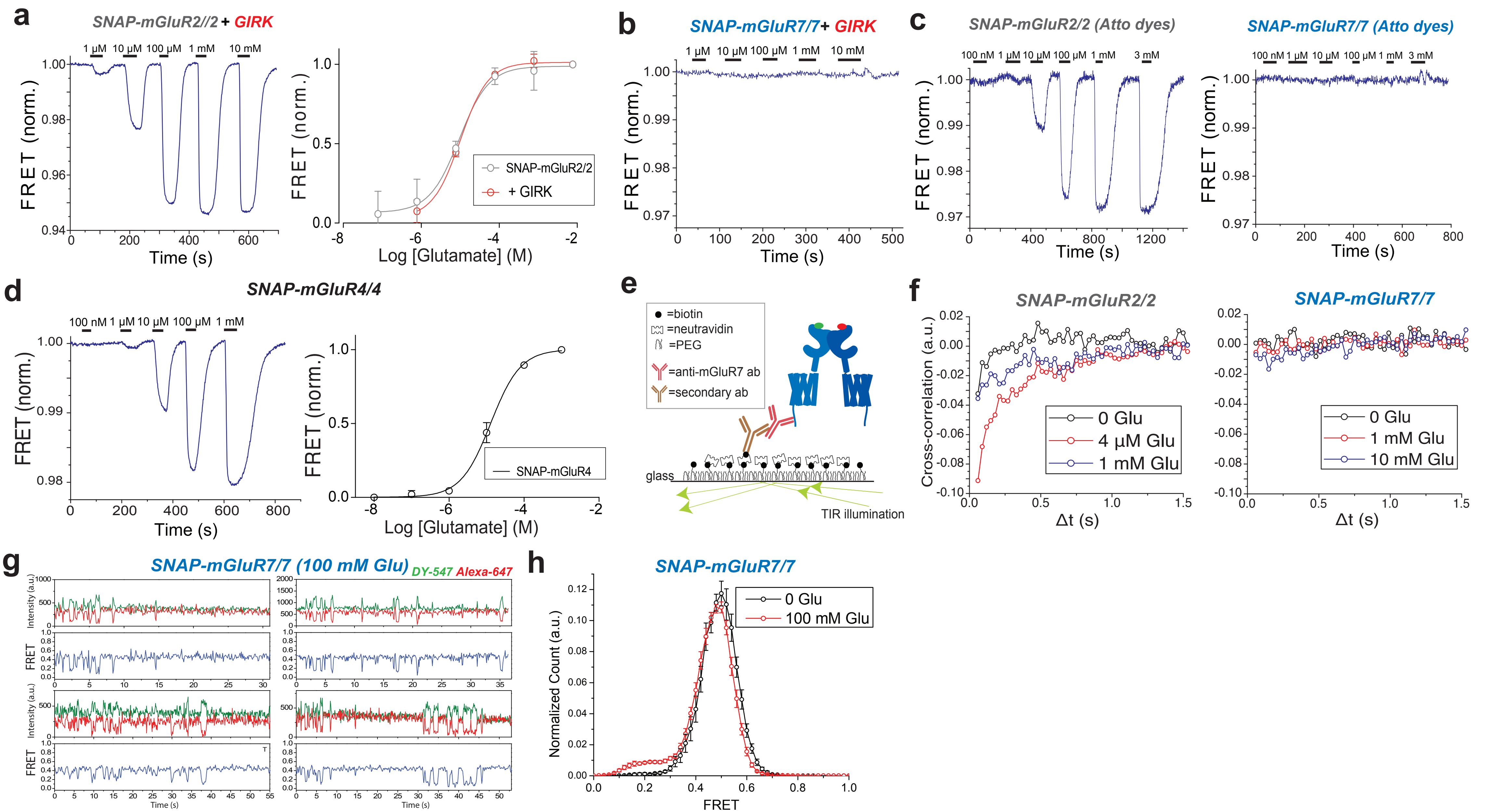

Supplemental Figure 1: Glutamate-induced FRET response of mGluR2/2, mGluR7/7 and mGluR4/4 homodimers.

(a-d) Live HEK293 cell ensemble FRET glutamate representative dose-response traces (a and d, left; b and c) and dose-response relations (a and d, right) for SNAP-mGluR2/2 co-expressed with GIRK, s.e.m error bars. (a); SNAP-mGluR7/7 co-expressed with GIRK (b); SNAP-mGluR2/2 (left) and SNAP-mGluR7/7 (right) (c); and SNAP-mGluR4/4, s.e.m. error bars (d). Receptors labeled with SNAP-selective, BG dyes: DY-547 (donor) and Alexa 647 (acceptor) (a,b,d) or Atto-488 (donor) and Atto-594 (acceptor) (c), imaged at 10 Hz.

(e) Schematic of individual dimer antibody pulldown and TIRF imaging of solubilized receptor.

(f) Donor/acceptor fluorescence cross-correlation for SNAP-mGluR2/2 (left) and SNAP-mGluR7/7 (right) at different glutamate concentrations shows greater dynamics in mGluR2/2 at 4 μM glutamate (4 movies, 311 molecules), near the EC50 where receptors transition most frequently between resting (high FRET) and activated (low FRET) states, than seen in 10 mM glutamate, the foot of the mGluR7/7 dose-response relation.

(g) Single molecule donor (green), acceptor (red) and FRET (blue) traces of SNAP-mGluR7/7 in 100 mM glutamate.

(h) Histograms from distinct data set from Fig. 1f of SNAP-mGluR7/7 in 0 (6 movies, 254 molecules, s.e.m. error bars) and 100 mM (6 movies, 308 molecules, s.e.m. error bars) glutamate.

(f-h) smFRET donor (BG-DY-547) and acceptor (BG-Alexa 647) dyes imaged at 10 Hz.

Supplemental Figure 2

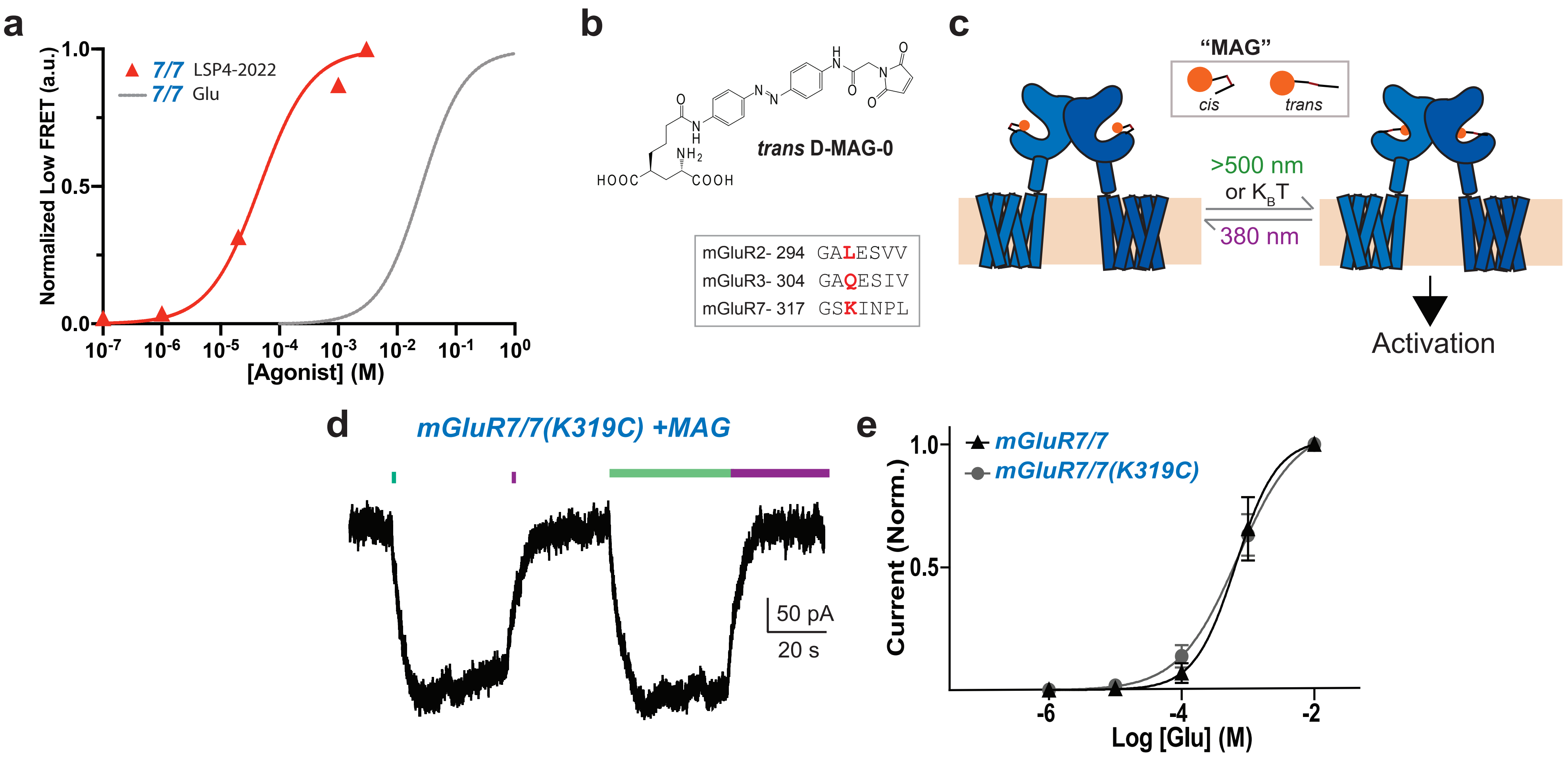

Supplemental Figure 2: LSP4-2022 and D-MAG activation of mGluR7/7.

(a) LSP4-2022 dose-response relation (red symbols and single Boltzmann fit) compared to that for glutamate (grey single Boltzmann fit from Fig. 1f) of low FRET (activated state) peak from smFRET histograms of SNAP-mGluR7. Donor (BG-DY-547) and acceptor (BG-Alexa 647) dyes imaged at 10 Hz.

(b) *Trans* D-MAG-0 structure (top) and amino acid sequence in lower lobe of three mGluRs around site of cysteine substitution for D-MAG-0 attachment (bottom, red single letter amino acid code).

(c) Schematic of mGluR7(K319C) homodimer photoactivation in *trans* configuration of D-MAG-0.

(d) Whole cell patch clamp recording in HEK293 cell of GIRK current activated by photoisomerization of D-MAG-0 on mGluR7(K319C) to *trans* with 532 nm light (green bars) and deactivated with 380nm light (violet vertical bars).

(e) Glutamate dose-response of GIRK current activation by wildtype mGluR7 (wt) same as by mGluR7(K319C), s.e.m. error bars

Supplemental Figure 3

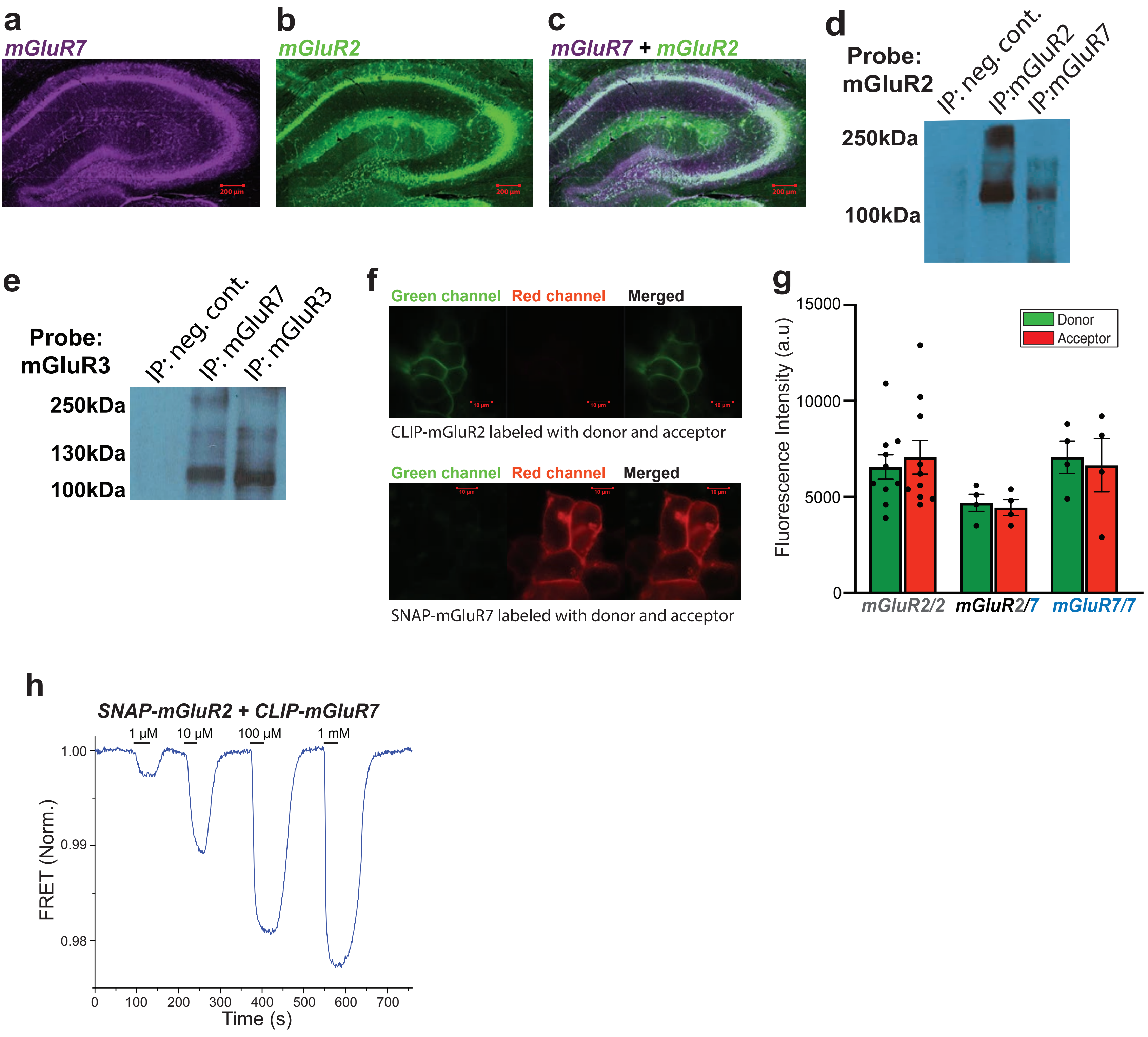

Supplemental Figure 3: mGluR2 and mGluR7 associate in hippocampus and assemble into a functional heterodimer in HEK293 cells.

(a-c) Adult mouse hippocampus section labeled with antibodies against mGluR7 (a and c, violet) and mGluR2 (b and c, green) with overlap indicated (c), white. Scale bar 200 µm.

(d) Immune precipitation from adult rat hippocampus with either anti-mGluR2 antibody (middle lane) or anti-mGluR7 antibody (right lane) probed with anti-mGluR2 antibody shows that mGluR2 is associated with mGluR7. The negative control (left lane) is immune precipitation without primary antibody.

(e) Immune precipitation from adult rat cortex with either anti-mGluR7 antibody (ab53705, middle lane) or anti-mGluR3 antibody (NBP2-61843, right lane) probed with anti-mGluR3 antibody shows that mGluR3 is associated with mGluR7. The negative control (left lane) is immune precipitation without primary antibody.

(f) HEK293 cells expressing CLIP-mGluR2 are selectively labeled by donor BC-549 (green), whereas cells expressing SNAP-mGluR7 are selectively labeled by acceptor BG-Alexa-647 (red). Scale bars 10 µm.

(g) Donor and acceptor fluorescence intensities are similar in: SNAP-mGluR2/SNAP-mGluR2 and SNAP-mGluR7/SNAP-mGluR7 labeled with donor BG-549 (green) and acceptor BG-Alexa-647 (red), and CLIP-mGluR2/SNAP-mGluR7 labeled with donor BC-549 (green) and acceptor BG-Alexa-647 (red). Individual data points (black dots) and s.e.m. (error bars).

(h) Representative live-cell FRET trace of SNAP-mGluR2/CLIP-mGluR7 heterodimer in response to ascending concentrations of glutamate.

Supplemental Figure 4

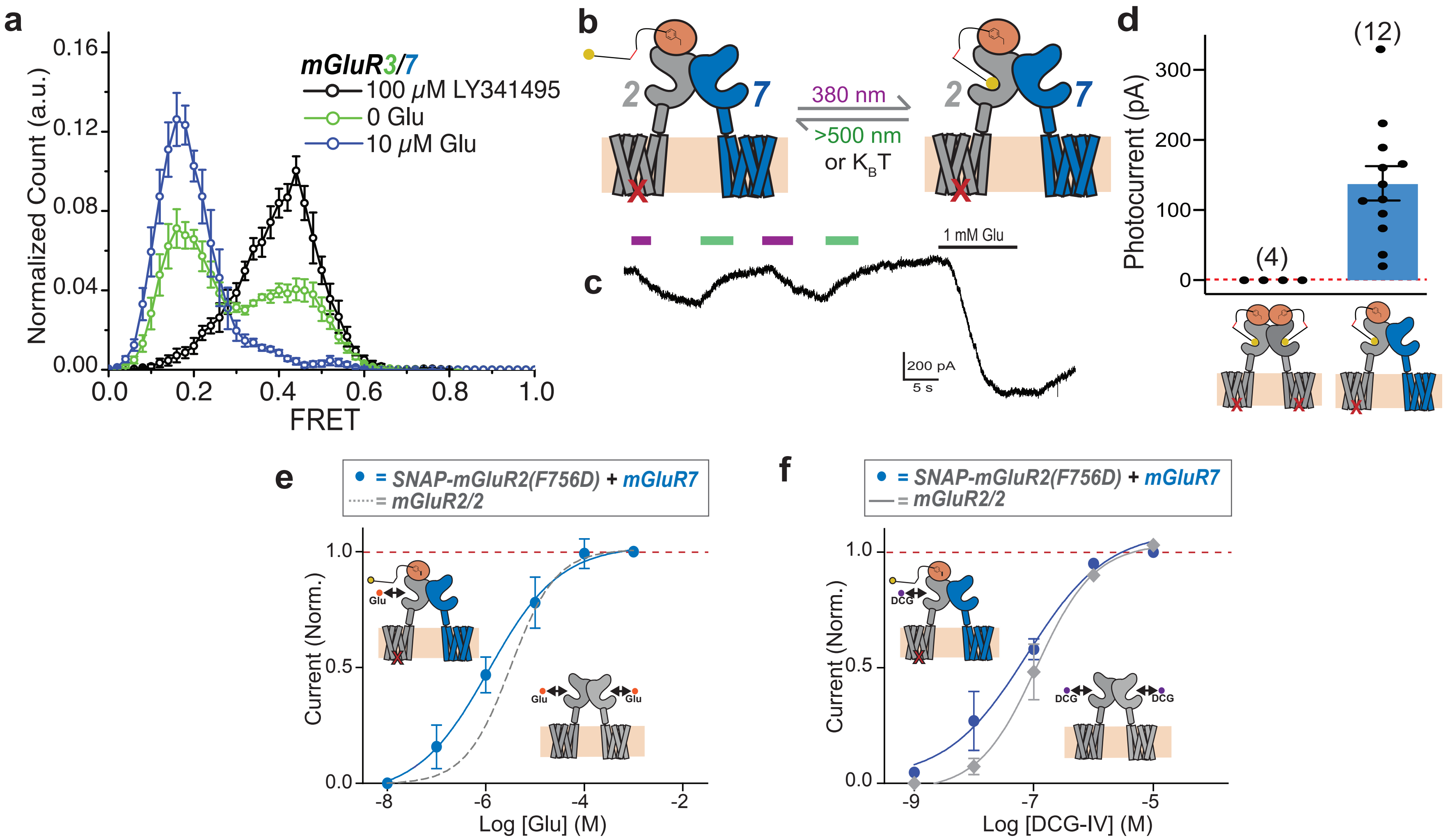

Supplemental Figure 4: mGluR2/7 enhanced affinity in effector activation.

(a) smFRET histograms of CLIP-mGluR3/SNAP-mGluR7 labeled with BC-549 and BG-Alexa-647 at 0 glutamate (green symbols, 7 movies, 298 molecules, s.e.m. error bars), 10 μM glutamate (blue symbols, 7 movies, 217 molecules, s.e.m. error bars) and 100 μM LY341495 (black symbols, 4 movies, 120 molecules, s.e.m. error bars).

(b) Schematic of mGluR2/7 heterodimer containing a signaling-dead SNAP-mGluR2(F756D) (containing a mutation at the G protein binding site, red X) and a wildtype mGluR7. The Photoswitched Orthogonal Remotely Tethered Ligand (PORTL) BGAG attaches to the SNAP tag on mGluR2(F756D) and signals to effector via trans-activation of the signaling-competent mGluR7.

(c) Whole cell patch clamp trace of SNAP-mGluR2(F756D)/mGluR7 co-expressed with GIRK1(F137S), which forms homotetrameric channels, in HEK293. The SNAP tag of SNAP-mGluR2(F756D) is labeled with BGAG and photo-activated by 380 nm light (violet bars) and photo-deactivated by 532 nm light (green bars), yielding reversible activation of inward GIRK current.

(d) No photocurrent detected in GIRK1(F137S) expressing HEK293 cells co-expressing just SNAP-mGluR2(F756D), but substantial photocurrent in cells co-expressing SNAP-mGluR2(F756D) and mGluR7. Numbers above bars indicate number of cells tested. Individual data points (black dots) and s.e.m. (error bars).

(e) Glutamate dose-response relation of SNAP-mGluR2(F756D)/mGluR7 (blue symbols and solid blue single Boltzmann fit) is left-shifted compared to that of mGluR2/2 (grey dashed single Boltzmann fit), s.e.m. error bars.

(f) DCG-IV dose-response relation of SNAP-mGluR2(F756D)/mGluR7 (blue symbols and solid blue single Boltzmann fit) is left-shifted compared to that of mGluR2/2 (grey symbols and solid grey single Boltzmann fit), s.e.m. error bars

Supplemental Figure 5

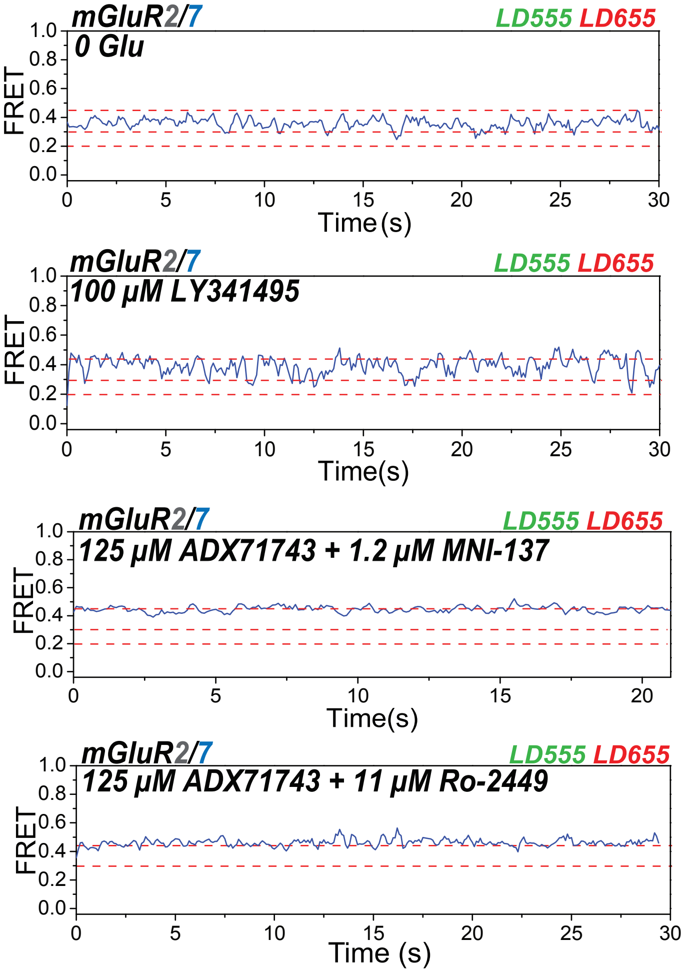

Supplemental Figure 5: Spontaneous rearrangements in mGluR2/7.

Representative smFRET trace of CLIP-mGluR2/SNAP-mGluR7 in 0 glutamate (top), 0 glutamate + 100 μM of the orthosteric antagonist LY341495 (middle), 0 glutamate + 1.2 μM MNI-137 + 125 μM ADX 71743, the mGluR2 and mGluR7 negative allosteric modulators, respectively (bottom). BC-DY-547 (donor) and BG-Alexa-647 (acceptor) imaged at 10 Hz.

Supplemental Figure 6

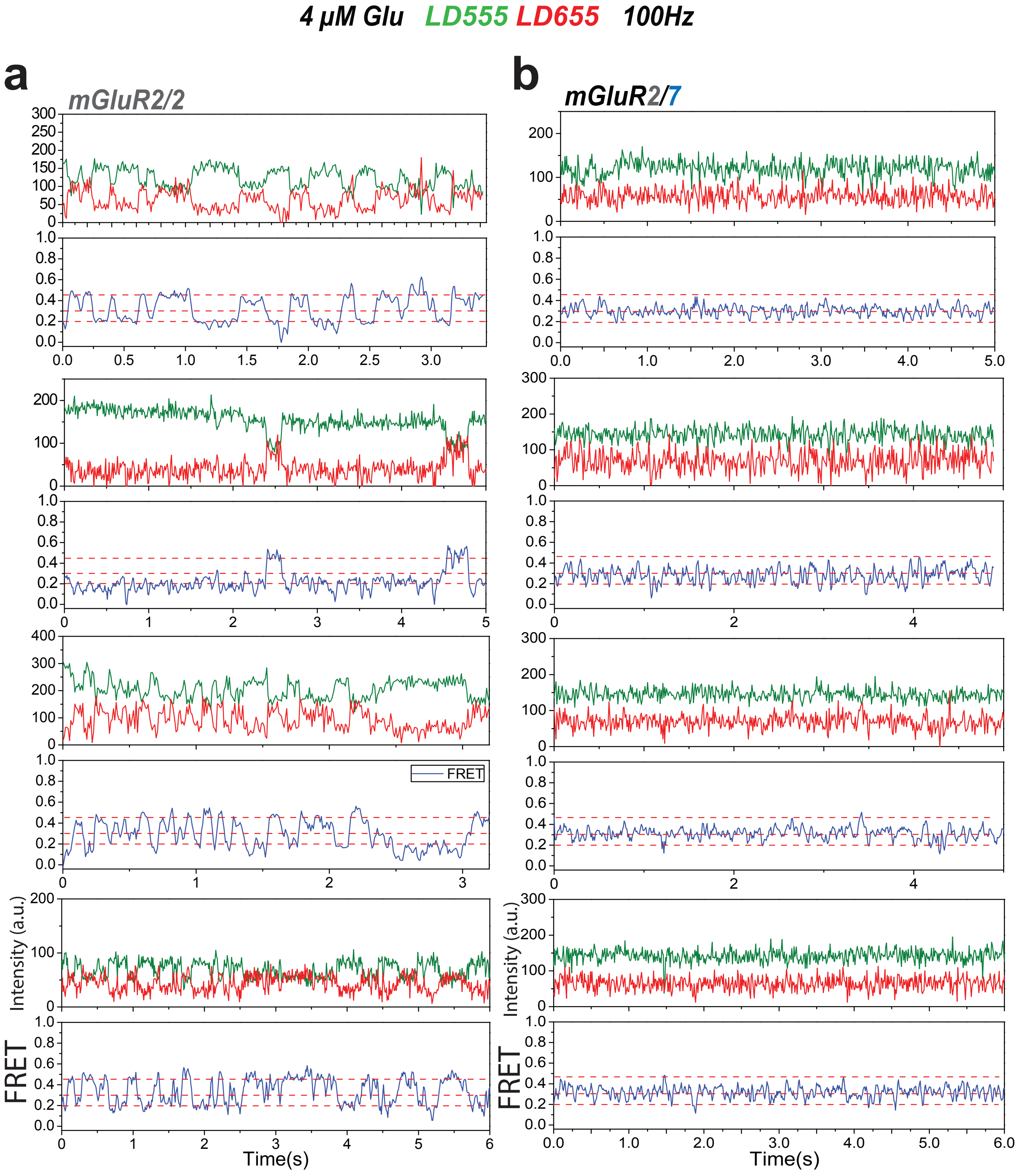

Supplemental Figure 6: Distinct LBD conformational dynamics in mGluR2/2 and mGluR2/7.

SNAP-mGluR2/SNAP-mGluR2 (mGluR2/2) (a) and CLIP-mGluR2/SNAP-mGluR7 (mGluR2/7) (b) in 4μM glutamate show that mGluR2/2 toggles between long occupancy high and low FRET states and mGluR2/7 spends most of the time in the intermediate FRET state, with brief excursions to the high or low FRET states. Donor BC-LD555 or BG-LD555 (green traces), acceptor BG-LD655 (red traces) and FRET (blue traces) imaged at 100 Hz.
